# Beyond device parameters: psychological predictors of spinal cord stimulation success and their neurobiological mechanisms—a narrative review

**DOI:** 10.3389/fpsyg.2026.1783577

**Published:** 2026-05-13

**Authors:** Anna B. Marcinkowska, Jakub Wiśniewski, Michał Sobstyl, Sara Kierońska-Siwak, Paweł J. Winklewski

**Affiliations:** 1 Applied Cognitive Neuroscience Lab, Department of Neurophysiology, Neuropsychology and Neuroinformatics, Medical University of Gdańsk, Gdańsk, Poland; 2 2nd Department of Radiology, Medical University of Gdańsk, Gdańsk, Poland; 3 Department of Neurosurgery, Copernicus Hospital in Gdansk, Gdańsk, Poland; 4 Department of Neurosurgery, Institute of Psychiatry and Neurology, Warsaw, Poland; 5 Department of Clinical Pathomorphology, Faculty of Medicine, Collegium Medicum in Bydgoszcz, Nicolaus Copernicus University in Torun, Bydgoszcz, Poland; 6 Department of Neurophysiology, Neuropsychology and Neuroinformatics, Medical University of Gdańsk, Gdańsk, Poland

**Keywords:** chronic neuropathic pain, neurobiological mechanisms, pain catastrophizing, psychological assessment, spinal cord stimulation

## Abstract

Spinal cord stimulation (SCS) represents an established treatment for refractory chronic pain, yet significant outcome variability persists despite technological advances. This narrative review examines why psychological factors assume central importance in neuromodulation outcomes by establishing pathophysiological contrasts between SCS and pharmacological pain management. Unlike passive pharmaceutical approaches operating through systemic biochemical modulation, SCS requires active patient engagement in device operation, expectancy management, and behavioral adaptation, creating distinct neurobiological requirements for success. Multiple psychological domains predict outcomes. Cognitive factors include pain catastrophizing (the strongest predictor), patient expectations, and cognitive control capacity. Emotional determinants encompass pre-implantation depression, anxiety, and alexithymia. Behavioral patterns such as fear-avoidance, locus of control, and coping strategies significantly influence treatment success. Neuroimaging evidence suggests that these psychological factors modulate response through alterations in prefrontal–limbic circuits, reward processing networks, and cortical reorganization. Maladaptive profiles are associated with reduced prefrontal top-down control, heightened limbic reactivity, impaired mesolimbic reward valuation, and persistent central sensitization. As a narrative synthesis prioritizing conceptual integration over quantitative aggregation, this review: (1) establishes psychological readiness as a neurobiological prerequisite rather than an administrative requirement, (2) delineates mechanisms through which catastrophizing, depression, and fear-avoidance disrupt SCS efficacy via limbic hyperactivity and impaired descending modulation, and (3) presents evidence-based optimization strategies including pre-implantation cognitive-behavioral therapy, acceptance and commitment therapy, behavioral activation, graded exposure, and digital health technologies. Future directions require standardized multidomain assessment protocols, multimodal predictive models integrating psychological phenotypes with objective biomarkers, and expanded digital therapeutics maintaining evidence-based rigor.

## 1 Introduction

Spinal cord stimulation (SCS) is an evidence-based interventional treatment for chronic neuropathic pain refractory to conservative therapies. Electrodes implanted epidurally deliver electrical impulses that modulate pain transmission before signals reach the brain (Melzack and Wall, 1965). Modern systems offer various stimulation modes, including paresthesia-free high-frequency (10 kHz), Burst™, and Fast Acting Sub-Perception (FAST™), providing flexibility in pain management (Campbell et al., 2013; Shanthanna et al., 2023). Primary indications include persistent spinal pain syndrome types 1 and 2, complex regional pain syndrome, and diabetic neuropathy (Taylor et al., 2014). Before permanent implantation, patients undergo a trial phase, with efficacy typically defined as ≥50% pain reduction. Long-term studies demonstrate sustained analgesia, functional improvement, and enhanced quality of life (Kumar et al., 2007). Beyond chronic pain management, this psychological-neural framework extends to spinal cord injury (SCI), where neuropathic pain commonly co-occurs with autonomic dysfunction and psychological comorbidities (Siddall et al., 2003). Evidence shows spinal cord stimulation can address both pain and autonomic dysfunction in SCI (Carhart et al., 2004; Flett et al., 2022; Herrity et al., 2018), suggesting psychological factors may similarly moderate neuromodulation outcomes through cortical-autonomic networks in these populations.

Despite technological advances, significant outcome variability persists, revealing that device parameters alone do not determine treatment success (Goudman et al., 2022). Psychological screening has evolved from optional to mandatory under most insurance regulations, including Medicare’s National Coverage Determination. In a European study of 482 SCS candidates, pain improvement varied from 86% in patients without psychosocial risk factors to 60% in those with severe problems, even when clinical appropriateness scores were identical (Goudman et al., 2022).

Unlike systemic analgesics operating through biochemical mechanisms largely independent of patient cognition (Argoff, 2011; Yam et al., 2018), SCS depends on cortical reorganization (Flor et al., 1997), patient agency in device management (Wiech et al., 2006), and extended temporal dynamics (Kumar et al., 2007). These processes are profoundly influenced by cognitive-emotional states (Apkarian et al., 2011; Edwards et al., 2016b).

While previous reviews, notably Goudman et al. (2022) proposed structured decision-making models for SCS qualification, they primarily conceptualized psychological assessment as a risk stratification or exclusion tool. The present review advances the field by reframing psychological readiness as a neurobiological prerequisite for neuromodulation efficacy. By integrating neuroimaging, neurophysiological, and psychological evidence, we explicitly map how cognitive-emotional variables translate into altered cortical–subcortical circuits that directly modulate descending pain control. This framework provides clinicians with a mechanistic rationale for why psychological screening and prehabilitation are not administrative requirements but integral components of SCS treatment success.

## 2 Methods

This review aimed to address the following research question: Which psychological factors predict treatment outcomes in adult patients undergoing SCS for chronic or neuropathic pain?

### 2.1 Search strategy

A systematic literature search was conducted in PubMed and Scopus databases. The search strategy combined keywords related to spinal cord stimulation, chronic pain, and psychological predictors using Boolean operators:

(“spinal cord stimulation” OR “spinal cord stimulator” OR “neuromodulation”) AND (“chronic pain” OR “neuropathic pain”) AND (“psychological” OR “psychosocial” OR “emotional” OR “cognitive”) AND (“predictors” OR “determinants” OR “outcome” OR “treatment response”).

The search was limited to peer-reviewed articles published in English. Reference lists of eligible studies were manually screened to identify additional relevant publications.

The search strategy was designed to ensure comprehensive coverage of relevant literature rather than to support a fully systematic review process.

### 2.2 Eligibility criteria

#### 2.2.1 Inclusion criteria

Studies were included if they met all of the following criteria:

- Human studies involving adult patients (≥18 years) treated with SCS
- Assessment of psychological, psychosocial, emotional, cognitive, or behavioral variables
- Reporting associations between psychological factors and SCS treatment outcomes
- Observational (prospective or retrospective) or interventional study designs
- Peer-reviewed original research articles.

#### 2.2.2 Exclusion criteria

Studies were excluded if they met any of the following criteria:

- Narrative reviews, systematic reviews, meta-analyses, scoping reviews, or clinical guidelines
- Case reports or case series including fewer than 10 patients
- Studies without psychological assessment
- Studies not reporting SCS-related treatment outcomes
- Technical or engineering-focused studies addressing device parameters only
- Animal or *in vitro* studies.

### 2.3 Study selection

**Figure 1:**
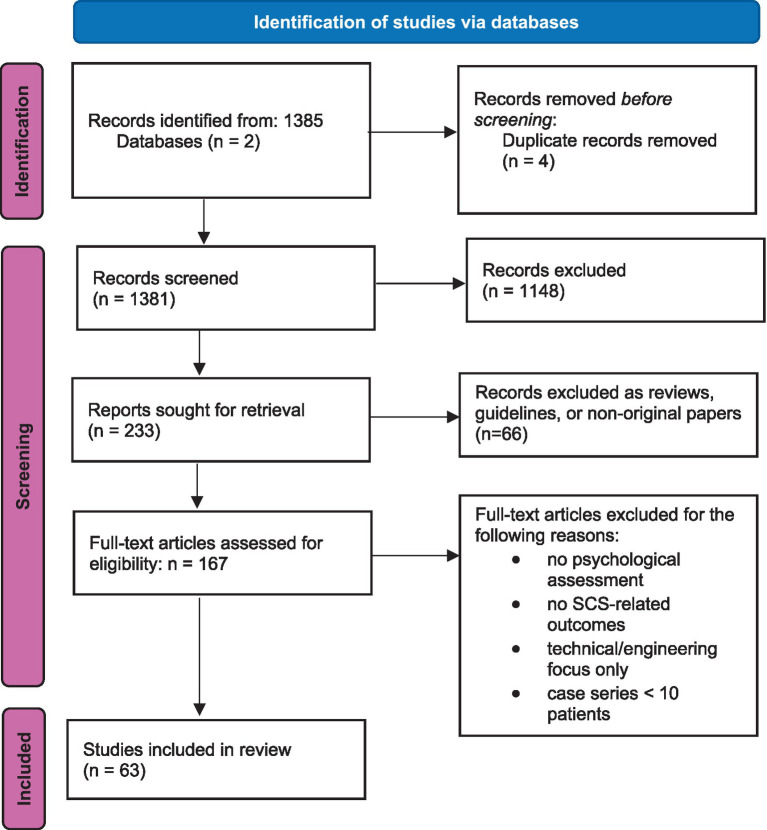
PRISMA-ScR flow diagram of study selection. The diagram illustrates the identification, screening, eligibility assessment, and inclusion of studies examining psychological predictors of treatment outcomes in spinal cord stimulation for chronic or neuropathic pain. Created in https://BioRender.com.

All records identified through database searches were screened based on titles and abstracts. Study screening and eligibility assessment were independently performed by two reviewers, with discrepancies resolved through discussion and consensus. Full-text articles were subsequently reviewed for eligibility. Study selection aimed to maximize coverage of clinically relevant psychological predictors while maintaining methodological transparency, consistent with the narrative review approach rather than strict systematic inclusion criteria; the selection process is summarized in a PRISMA-ScR flow diagram (Figure 1).

### 2.4 Data extraction

For each included study, the following data were extracted:

- Study design and sample size
- Type of chronic or neuropathic pain condition
- Psychological variables assessed
- Psychological assessment instruments used
- SCS-related outcomes measured (e.g., pain reduction, functional improvement, quality of life, device retention)
- Direction and strength of associations between psychological factors and treatment outcomes.

### 2.5 Quality considerations

Given the substantial heterogeneity of study designs, populations, psychological constructs, and outcome measures, a formal risk of bias assessment using standardized tools (e.g., ROBINS-I, Newcastle-Ottawa Scale) was not performed, consistent with the narrative review approach adopted here. The included studies varied widely, encompassing prospective observational studies, randomized controlled trials, and retrospective cohort analyses, which precluded the application of a single appraisal framework across methodologically incompatible designs. Methodological considerations such as sample size, direction and consistency of findings, and potential confounders were therefore evaluated qualitatively during data interpretation. The PRISMA-ScR flow diagram (Figure 1) is retained to ensure transparency of the literature search and selection process, rather than to imply formal systematic review methodology.

### 2.6 Data synthesis

Given the heterogeneity of study designs, psychological constructs, assessment instruments, and outcome measures, a quantitative meta-analysis was not feasible. Results were therefore synthesized using a structured narrative approach, emphasizing consistency of findings across studies and integration with neurobiological and clinical frameworks relevant to SCS. PRISMA-ScR flow diagram illustrating the identification, screening, eligibility assessment, and inclusion of studies examining psychological predictors of SCS outcomes.

## 3 Pathophysiological contrasts: spinal cord stimulation vs. classic pain management

Understanding how SCS differs mechanistically from pharmacological approaches clarifies why psychological factors assume central importance in neuromodulation outcomes. While both aim to reduce pain, they engage fundamentally different neural systems, creating divergent requirements for patient engagement.

Classic pharmacological pain management operates through systemic chemical modulation of nociceptive signaling. Opioids modulate mu, delta, and kappa receptors; NSAIDs inhibit prostaglandin synthesis; anticonvulsants modulate calcium channels; and antidepressants enhance descending inhibition. These systemic agents alter biochemical signaling throughout the nervous system with limited capacity to reverse maladaptive supraspinal reorganization (Argoff, 2011; Yam et al., 2018). Psychological factors influence treatment outcomes through placebo effects, adherence, and stress-inflammation interactions, yet pharmacological agents can produce analgesic effects with minimal patient engagement beyond medication taking.

In contrast, SCS delivers focal electrical impulses to the dorsal columns, modulating ascending nociceptive signals before they reach supraspinal centers. This operates via Gate Control Theory by activating large-diameter A-beta fibers that inhibit transmission of nociceptive signals in the spinothalamic tract (Kapural et al., 2016; Melzack and Wall, 1965). Modern high-frequency (10 kHz) and burst stimulation achieve paresthesia-free analgesia through mechanisms beyond simple gate control, including modulation of wide-dynamic-range neurons, GABAergic activation, and glial normalization (De Ridder et al., 2013; Lee et al., 2020). Recent evidence demonstrates that high-frequency SCS modulates cortical oscillations in somatosensory and association cortices, with suppression of high-frequency (70-150 Hz) activity and region-specific modulation of low-frequency oscillations, suggesting supraspinal mechanisms underlying analgesic effects (Bharmauria et al., 2025). Closed-loop systems utilizing evoked compound action potentials enable real-time adjustment based on neural responses. The ECHO-MAC trial demonstrated that evoked compound action potential-controlled stimulation reduced overstimulation by 97.6% compared with open-loop systems while maintaining efficacy (Mangano et al., 2025; Nijhuis et al., 2023; Will et al., 2024).

SCS requires sustained active patient engagement across multiple domains to achieve optimal outcomes. Patients must learn to operate programming devices, communicate effectively about stimulation quality and coverage, participate in systematic parameter optimization, adhere to battery charging requirements, attend frequent follow-up appointments, and most critically, leverage pain reduction for behavioral activation and functional restoration (Deer et al., 2014; Goudman et al., 2022). The subjective sense of agency, defined as the experience of controlling one’s device settings and treatment outcomes, activates prefrontal regions that exert descending pain inhibition, creating direct analgesic effects independent of stimulation parameters (Wiech et al., 2006).

## 4 The biopsychosocial model in SCS

Modern understanding of pain mechanisms extends beyond simple nociceptive transmission. Melzack and Wall’s Gate Control Theory introduced the revolutionary concept that pain perception involves complex modulation at spinal and supraspinal levels, with psychological factors influencing this “gate” (Melzack and Wall, 1965). While groundbreaking, this model underestimated the complexity of cortical contributions, inadequately explained chronic pain conditions persisting without ongoing peripheral input (such as phantom limb pain), and lacked compatibility with numerous neurophysiological findings (Moayedi and Davis, 2013). Melzack’s subsequent Neuromatrix Theory proposed that pain emerges from distributed brain networks integrating sensory, affective, and cognitive dimensions (Melzack, 2001), but remained anatomically vague with limited testable predictions.

Contemporary predictive coding models address these limitations by framing pain as Bayesian inference about tissue state based on sensory input, prior beliefs, and contextual factors (Büchel et al., 2014; Song et al., 2021). This framework explains how expectations, attention, and emotional state modulate pain through top-down influences on nociceptive processing (Chen and Wang, 2023; Mulders et al., 2023). Recent computational models demonstrate that pain perception involves hierarchical predictive coding across distributed circuits, with the cingulate-insula network serving as major hubs coordinating sensory and affective components (Chen, 2023; Mancini et al., 2022). However, the full applicability of predictive coding to acute nociceptive pain intensity remains under investigation.

Chronic pain involves maladaptive neuroplastic changes termed central sensitization, wherein the nervous system amplifies pain signals and reduces inhibitory control (Latremoliere and Woolf, 2009). Central Sensitization Inventory scores correlate more strongly with psychological factors (depression, anxiety, catastrophizing) than with psychophysical measures of pain amplification (Salbego et al., 2025; Takeuchi et al., 2024), suggesting central sensitization questionnaires may primarily capture psychological constructs rather than pure nociceptive sensitization.

## 5 Evidence of psychological predictors of SCS outcomes

Social support, including family encouragement, practical assistance, and emotional validation, facilitates rehabilitation and device utilization while buffering against depression. Realistic expectations that SCS reduces rather than eliminates pain predict greater satisfaction, with patients viewing partial relief as meaningful success (Linde et al., 2007). Strong self-efficacy promotes sustained engagement and problem-solving when complications arise, acting as a protective factor against catastrophizing and depression (Kardash et al., 2024; Solberg Nes et al., 2024). Previous participation in multidisciplinary treatment may reflect greater readiness for SCS therapy, including the ability to actively engage in treatment and understand the complex biopsychosocial nature of chronic pain (Goudman et al., 2022).

### 5.1 Cognitive factors

Pain catastrophizing amplifies attention to pain signals, triggers exaggerated threat appraisals, and undermines confidence in pain management (Quartana et al., 2009). Even with substantial nociceptive reduction, catastrophizers may fail to recognize improvement or attribute it to temporary device function rather than stable benefit. Attention bias maintains heightened awareness of pain-related stimuli, with patients continuously monitoring bodily sensations and interpreting ambiguous signals as pain recurrence, preventing the cognitive disengagement needed for functional restoration (Eccleston et al., 2014).

Patient expectations function as self-fulfilling prophecies, with realistic positive expectations enhancing outcomes while unrealistic expectations of complete cure predict disappointment through both neurobiological mechanisms and behavioral patterns (Linde et al., 2007). Cognitive control, the capacity to regulate attention, inhibit maladaptive thoughts, and engage in goal-directed behavior, facilitates effective device use and rehabilitation engagement (Turner et al., 2007).

### 5.2 Emotional and affective factors

Pre-implantation depression predicts reduced pain relief and poorer functional outcomes through overlapping neural circuits that amplify pain via altered monoaminergic systems, impaired descending inhibition, and reduced treatment engagement (Bair et al., 2003; Bernaerts et al., 2025; Fishbain et al., 1997).

Anxiety, particularly health anxiety and pain-related fear, promotes hypervigilance, catastrophic misinterpretation of sensations, and avoidance behaviors that undermine device utilization despite adequate pain relief (Asmundson and Katz, 2009; Klasova et al., 2025).

Alexithymia impairs communication with providers and engagement with psychological interventions, as patients struggle to distinguish pain from emotional distress, leading to somatization and reduced responsiveness to cognitive-behavioral strategies (Lumley et al., 2011).

Conversely, emotional regulation capacity represents a critical protective factor. Adaptive strategies such as cognitive reappraisal and acceptance predict better outcomes, while maladaptive patterns including emotional suppression and rumination predict worse results (McCracken and Vowles, 2014). Patients with active coping strategies and lower illness anxiety demonstrate superior long-term outcomes (Bernaerts et al., 2025).

### 5.3 Fear-avoidance and behavioral activation

The fear-avoidance model explains how catastrophic pain interpretations trigger protective avoidance behaviors that paradoxically worsen outcomes through physical deconditioning and enhanced pain sensitivity (Alaiti et al., 2025; Vlaeyen et al., 2016). High pre-implantation fear-avoidance and catastrophizing scores predict poorer functional outcomes even when adequate pain relief is achieved, as patients fail to leverage reduced pain for activity re-engagement (Celestin et al., 2009; Rosenberg et al., 2015).

Behavioral activation—systematic engagement in valued activities despite discomfort—disrupts avoidance cycles, while graded exposure therapy progressively confronts feared movements in controlled contexts (Ryum and Stiles, 2023; Simons et al., 2023). SCS-induced pain reduction creates a “window of opportunity” for behavioral activation that might otherwise be too threatening. This synergy between device-mediated analgesia and exposure-based psychological intervention represents a key mechanism through which pre-implant psychological preparation enhances outcomes.

### 5.4 Behavioral and motivational factors

Locus of control significantly influences treatment outcomes through patient engagement and self-management. Internal locus of control, the belief that outcomes depend on personal actions, predicts active engagement and superior results (Crisson and Keefe, 1988). These patients view SCS as a tool to optimize through participation, leading to persistent device adjustment and rehabilitation adherence. Conversely, external locus of control, attributing outcomes to chance or others’ actions, predicts passivity, with patients expecting the device alone to produce relief without behavioral change.

Active coping strategies, including problem-solving, maintained activity engagement, and cognitive reframing, consistently correlate with favorable outcomes by enabling patients to leverage pain reduction for functional restoration (Brown and Nicassio, 1987). Passive coping approaches such as behavioral withdrawal and complete delegation to medical providers predict poor outcomes even when adequate analgesia is achieved. Recent Belgian registry data confirmed that patients with more active and fewer passive coping strategies prior to SCS trial demonstrated superior long-term recovery and satisfaction (Bernaerts et al., 2025).

### 5.5 Social and contextual moderators

Supportive family relationships that encourage functional gains while validating suffering facilitate optimal outcomes through reinforcement of rehabilitation and emotional support (Goudman et al., 2022; Romano et al., 1992). Conversely, solicitous behaviors reinforcing disability or punishing responses worsen results by undermining motivation. Unemployment and disability benefit receipt are associated with poorer outcomes through loss of occupational identity, reduced daily structure, and social isolation rather than malingering (Kumar et al., 2006; North et al., 2005). Social support consistently predicts better outcomes by buffering against depression and reinforcing treatment engagement, while social isolation amplifies distress and predicts poor adherence (Bernaerts et al., 2025; Jamison and Virts, 1990).

### 5.6 Composite risk models

Individual psychological factors combine synergistically rather than additively to influence outcomes (Goudman et al., 2022). A patient with moderate depression and moderate catastrophizing may experience substantially worse outcomes than predicted by either factor alone, as these vulnerabilities interact to amplify pain perception and undermine coping. Composite assessment tools integrating multiple psychological domains demonstrate superior predictive validity compared to single-factor approaches.

Block’s Presurgical Psychological Screening methodology exemplifies this integrative approach, combining MMPI-2 profiles, catastrophizing scores, and psychosocial risk factors into weighted algorithms. Validation studies demonstrate that high-risk patients experience failure rates 3-4 times higher than low-risk patients (Deer et al., 2014; Goudman et al., 2022).

## 6 Neurobiological mechanisms

Understanding the neurobiological substrates linking psychological factors to SCS outcomes provides mechanistic grounding for clinical observations. Contemporary research reveals deep integration between cognitive-emotional processes and neural circuits that directly modulate pain perception and treatment response. This section examines how psychological mechanisms manifest in measurable brain activity patterns predicting SCS efficacy.

### 6.1 Neural circuits mediating expectancy effects

Positive expectations activate endogenous opioid, dopaminergic, and endocannabinoid systems producing measurable analgesia (Wager et al., 2013). Expectancy-induced analgesia preferentially modulates prefrontal cortex, anterior cingulate cortex, and reward regions rather than sensory areas (Botvinik-Nezer et al., 2024; Zunhammer et al., 2021), targeting pain’s affective-motivational dimension (how bothersome) over its sensory-discriminative aspects (location, intensity). The prefrontal-cingulate network exerts descending control onto brainstem periaqueductal gray and rostral ventromedial medulla, modulating spinal nociception. Pre-implantation expectations robustly predict SCS outcomes (Shanthanna et al., 2023), providing mechanistic rationale for psychological preparation.

### 6.2 Self-efficacy and prefrontal modulation

Self-efficacy activates dorsolateral and ventrolateral prefrontal regions implementing descending pain inhibition (Mosch et al., 2023). Perceived control directly reduces both subjective pain intensity and objective neural responses to identical stimuli (Mosch et al., 2023; Wiech, 2016). Chronic pain disrupts this mechanism: fibromyalgia patients fail to activate prefrontal modulation circuits, showing instead increased amygdala and parahippocampal activity (Mosch et al., 2023). Low self-efficacy may reduce top-down pain modulation capacity even with successful spinal SCS. Self-efficacy strongly predicts outcomes and protects against catastrophizing (Kardash et al., 2024; Solberg Nes et al., 2024).

### 6.3 Emotional regulation and central sensitization

Cognitive reappraisal engages ventrolateral and dorsolateral prefrontal regions modulating subcortical processing in amygdala and insula (Liu et al., 2023; Monachesi et al., 2023), reducing pain through dampening inflammation, enhancing descending inhibition, and preventing maladaptive neuroplasticity (Moriarity et al., 2023). The periaqueductal gray–rostral ventromedial medulla circuit either facilitates or inhibits spinal nociception depending on cognitive-emotional context (Ji et al., 2018). Effective regulation maintains inhibitory mode; maladaptive strategies shift toward facilitation.

### 6.4 Structural and functional brain alterations

Maladaptive patterns (catastrophizing, depression, anxiety, low self-efficacy) manifest in measurable alterations: reduced prefrontal gray matter, diminished prefrontal-limbic connectivity, heightened amygdala reactivity, and altered pain network dynamics (Malfliet et al., 2017; Seminowicz and Moayedi, 2017). These changes amplify pain perception independently of peripheral input and impair descending inhibitory pathways, potentially limiting SCS responsiveness despite technically successful spinal modulation.

### 6.5 Neurophysiological biomarkers and cortical oscillations

SCS modulates cortical oscillatory activity, particularly theta–gamma coupling patterns. Resting-state EEG reveals specific signatures: chronic back pain patients show positive association between ongoing pain intensity and prefrontal beta/gamma oscillations (May et al., 2019), reflecting subjective perception rather than objective nociceptive processing. Large-scale studies demonstrate increased theta (4-8 Hz) and gamma (>60 Hz) connectivity in frontal areas with global network reorganization (Ta Dinh et al., 2019), reflecting impaired top-down control associated with catastrophizing and low self-efficacy. These patterns support the thalamocortical dysrhythmia model.

### 6.6 Cerebellar contributions

Beyond motor coordination, the cerebellum integrates sensory, cognitive, and affective pain dimensions through extensive connectivity with dorsolateral prefrontal cortex, anterior cingulate cortex, insula, and posterior parietal cortex (Buckner, 2013; Moulton et al., 2010). Its predictive function relates to catastrophizing (excessive negative prediction via cerebello-cerebral circuits) and fear-avoidance (cerebellar encoding of movement-pain associations). Cerebellar inhibitory output via Purkinje cells suggests involvement in descending pain modulation (Moulton et al., 2010). SCS may indirectly influence cerebellar function through altered ascending/descending signals, suggesting psychological optimization strategies exert effects through multiple parallel neural pathways including cerebellar-mediated prediction and learning mechanisms.

### 6.7 Integration

**Figure 2:**
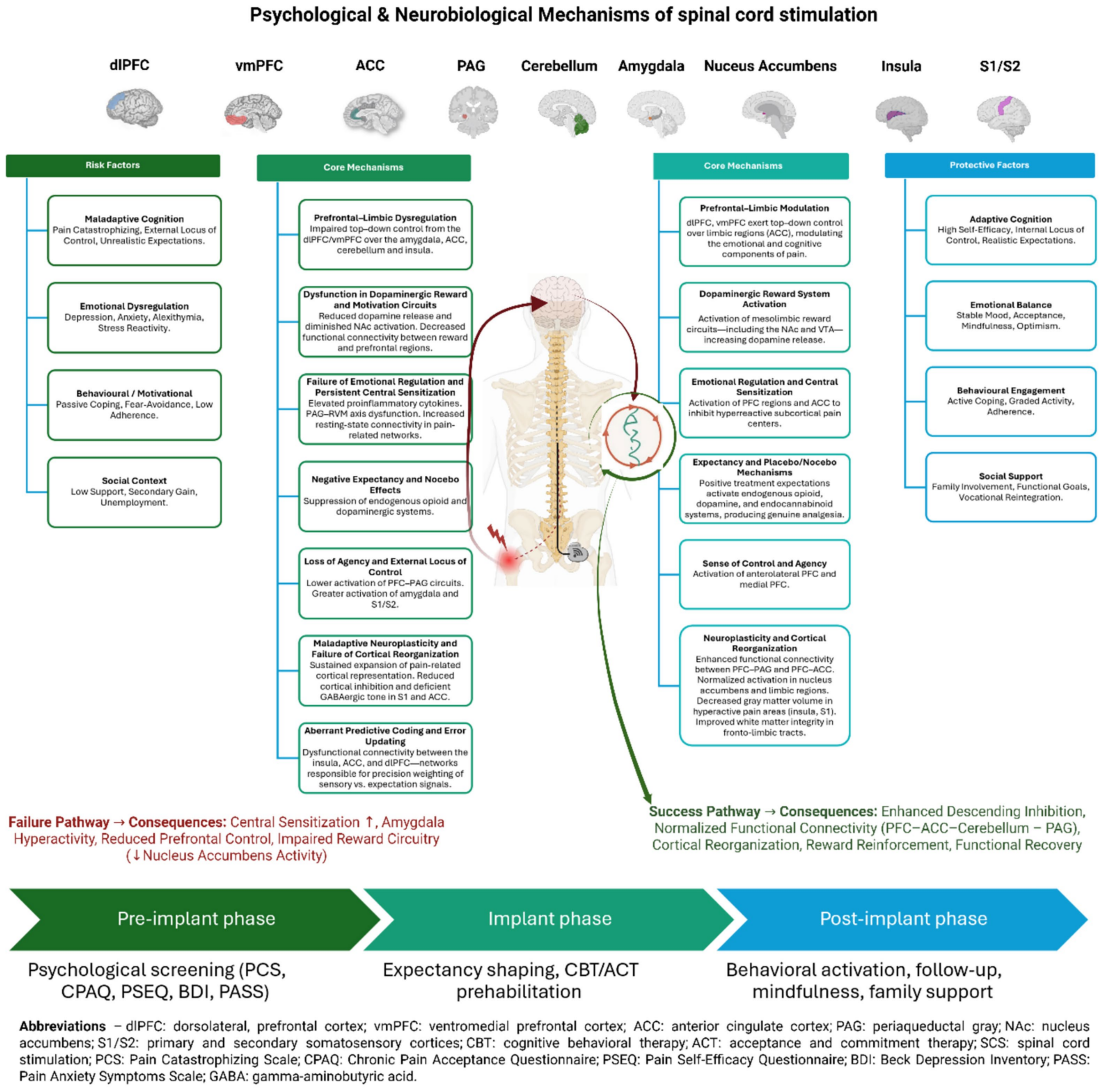
Psychological and neurobiological mechanisms underlying spinal cord stimulation outcomes. Left panel (Failure pathway). The failure pathway illustrates maladaptive cognitive–emotional factors, including pain catastrophizing, depressive symptoms, unrealistic expectations, and external locus of control, which disrupt prefrontal top-down modulation. This dysregulation is associated with increased limbic reactivity (amygdala, ACC), impaired reward processing (reduced nucleus accumbens activity), aberrant predictive coding, and persistent central sensitization. Dysfunctional connectivity within prefrontal–limbic–cerebellar networks further contributes to maladaptive pain prediction, fear-avoidance learning, and failure of cortical reorganization. Right panel (Success pathway). The success pathway depicts adaptive psychological and neurobiological mechanisms, including self-efficacy, emotional regulation, and realistic expectations, which enhance coordinated activity within prefrontal–limbic–cerebellar–brainstem circuits (dlPFC/vmPFC–ACC–cerebellum–PAG–RVM). These processes facilitate descending pain inhibition, normalize reward system function (VTA–NAc), support neuroplasticity and cortical reorganization within somatosensory cortices (S1/S2), and promote functional recovery following SCS. Bottom panel (Clinical timeline). The lower panel presents the clinical trajectory of SCS treatment, integrating pre-implant psychological screening (PCS, CPAQ, PSEQ, BDI, PASS), pre-implant psychological optimization and expectancy shaping (CBT/ACT), and post-implant consolidation through behavioral activation, mindfulness-based strategies, follow-up, and social support. Created in https://BioRender.com.

Evidence reveals coherent mechanistic pathway: psychological factors (expectations, self-efficacy, emotional regulation) → neural circuits (prefrontal-limbic-brainstem-cerebellar networks) → descending modulation → clinical SCS response. SCS modulates both spinal and supraspinal pain processing, but efficacy depends critically on functional integrity of descending control systems shaped by psychological factors. Patients with maladaptive patterns show neural signatures (reduced prefrontal activity, heightened limbic reactivity, altered brainstem/cerebellar modulation, dysregulated theta-gamma coupling) potentially limiting capacity to benefit from technically successful SCS (Figure 2, Table 1).

**Table 1:** Brain structures involved in pain perception and modulation relevant to spinal cord stimulation (SCS).

Structure/system	Functional role in pain	Specific relevance to SCS mechanisms	Key references
Dorsolateral Prefrontal Cortex (dlPFC)	Cognitive control, attentional modulation, top-down regulation	Enhances descending inhibition; mediates effects of expectancy and perceived control	Joyce et al. (2025), Seminowicz and Moayedi (2017)
Ventromedial Prefrontal Cortex (vmPFC)	Emotional regulation, valuation, threat appraisal	Modulates limbic responses and contributes to placebo/expectancy-related analgesia	Buhle et al. (2014), Joyce et al. (2025)
Anterior Cingulate Cortex (ACC)	Affective component of pain, conflict monitoring	Engaged during expectancy-induced analgesia; part of salience network integrating emotional pain signals	Buhle et al. (2014), Malfliet et al. (2017)
Insula (incl. Dorsal posterior insula)	Interoception, encoding of pain intensity and salience	Central node for subjective pain perception; integrates nociceptive input with cognitive–affective state	Buhle et al. (2014), Liu et al. (2023)
Amygdala	Fear, threat detection, emotional amplification of pain	Hyperactive in catastrophizing; contributes to amplified pain experience and impaired modulation	Buhle et al. (2014), Liu et al. (2023), Seminowicz and Moayedi (2017)
Hippocampus	Contextual memory, emotional learning	Altered in chronic pain; involvement in pain memory and contextual modulation impacting SCS outcomes	Seminowicz and Moayedi (2017)
Periaqueductal Gray (PAG)	Initiation of descending pain inhibition	Activated by expectations and self-efficacy; major hub where psychological factors converge with SCS-induced modulation	Ji et al. (2018)
Rostral Ventromedial Medulla (RVM)	Facilitates or inhibits spinal nociception	Works jointly with PAG to modulate spinal dorsal horn responses to stimulation	Ji et al. (2018)
Spinal Dorsal Horn	Primary gating of ascending nociceptive signals	Direct site of SCS action; stimulation activates inhibitory interneurons reducing nociceptive transmission	Melzack and Wall (1965)
Nucleus Accumbens (NAcc)	Reward processing, motivation, relief valuation	Critical for reinforcing analgesic effects; affected by depression and catastrophizing	Seminowicz and Moayedi (2017)
Ventral Tegmental Area (VTA)	Dopaminergic reward pathway	Supports motivation and engagement; dysfunction reduces benefit from SCS	Seminowicz and Moayedi (2017)
Primary Somatosensory Cortex (S1)	Somatotopic representation of sensory input	Demonstrates maladaptive reorganization in chronic pain; SCS may partially normalize maps	Seminowicz and Moayedi (2017)
Secondary Somatosensory Cortex (S2)	Sensory discrimination and integration	Participates in processing altered sensory input following stimulation	Seminowicz and Moayedi (2017)
Default Mode Network (DMN)	Self-referential thought, rumination	Overactive in catastrophizing; contributes to chronic pain persistence	Malfliet et al. (2017)
Salience Network (ACC + insula)	Detection of relevant sensory/emotional stimuli	Central for interpreting nociceptive signals and integrating affective meaning	Buhle et al. (2014), Malfliet et al. (2017)
Executive Control Network (dlPFC-centered)	Regulation of cognition and behavior	Supports adaptive coping and engagement with SCS therapy	Joyce et al. (2025), Seminowicz and Moayedi (2017)
Cerebellum	Sensory prediction, associative learning, cognitive–affective integration of pain	Supports maladaptive pain prediction and fear-avoidance via cerebello–cerebral circuits; may contribute to descending pain modulation and be indirectly influenced by SCS through altered ascending and descending signaling.	Buckner (2013), Moulton et al. (2010)

dlPFC, dorsolateral prefrontal cortex; vmPFC, ventromedial prefrontal cortex; ACC, anterior cingulate cortex; PAG, periaqueductal gray; RVM, rostral ventromedial medulla; NAcc, nucleus accumbens; VTA, ventral tegmental area; S1, primary somatosensory cortex; S2, secondary somatosensory cortex; DMN, default mode network; SCS, spinal cord stimulation; WDR, wide dynamic range (neurons); DNIC, diffuse noxious inhibitory controls.

The failure pathway illustrates maladaptive cognitive–emotional factors, including pain catastrophizing, depressive symptoms, unrealistic expectations, and external locus of control, which disrupt prefrontal top-down modulation. This dysregulation is associated with increased limbic reactivity (amygdala, ACC), impaired reward processing (reduced nucleus accumbens activity), aberrant predictive coding, and persistent central sensitization. Dysfunctional connectivity within prefrontal–limbic–cerebellar networks further contributes to maladaptive pain prediction, fear-avoidance learning, and failure of cortical reorganization.

## 7 Recommendations for pre-implantation assessment guidelines

Comprehensive pre-implantation psychological assessment identifies risk factors predicting poor outcomes, detects protective factors that can be fostered, and optimizes modifiable psychological variables. While SCS addresses spinal nociceptive transmission, treatment success depends on how patients cognitively interpret, emotionally respond to, and behaviorally engage with their modified pain experience.

### 7.1 Core assessment domains and instruments

As illustrated in Figure 2, psychological constructs assessed during pre-implantation screening map onto specific cortical–limbic–brainstem circuits that directly modulate spinal nociceptive processing and behavioral engagement with SCS therapy. Comprehensive evaluation for SCS candidacy should systematically assess multiple psychological domains using validated, standardized instruments (Celestin et al., 2009; Doleys, 2006). The following assessment framework provides evidence-based guidance for clinical practice (Table 2).

**Table 2:** Psychological constructs, assessment tools and their neurobiological relevance for spinal cord stimulation (SCS).

Psychological domain/construct	Assessment tool	Key CNS structures/networks	Neurobiological mechanism relevant to SCS	Clinical relevance for SCS qualification and optimization	Key references
Pain catastrophizing	Pain Catastrophizing Scale (PCS)	dlPFC, ACC, insula, amygdala, DMN	Reduced top-down inhibitory control, limbic hyperreactivity, increased salience attribution to nociceptive input, impaired engagement of descending pain modulation	Strongest predictor of poor SCS outcome; high scores indicate need for pre-implant CBT/ACT targeting threat appraisal and attentional bias	Malfliet et al., (2017) Quartana et al. (2009), Rosenberg et al. (2015)
Pain self-efficacy/perceived control	Pain Self-Efficacy Questionnaire (PSEQ)	dlPFC, vmPFC, PAG–RVM axis	Enhanced perceived agency activates prefrontal control networks and descending inhibitory pathways facilitating neuromodulation efficacy	Protective factor; low self-efficacy suggests benefit from preparatory psychological intervention enhancing agency and device engagement	Solberg Nes et al. (2024), Wiech et al. (2006)
Depressive symptoms	Beck Depression Inventory-II (BDI-II)	vmPFC, NAc, VTA, limbic network	Blunted reward processing, reduced motivation, impaired reinforcement learning, weakened descending inhibition	Predictor of reduced pain relief and higher explantation risk; treatable risk factor rather than absolute contraindication	Bair et al. (2003), Seminowicz and Moayedi (2017)
Anxiety/pain-related fear	Hospital Anxiety and Depression Scale (HADS); Tampa Scale for Kinesiophobia (TSK)	Amygdala, insula, ACC, salience network	Hypervigilance, exaggerated threat detection, maintenance of avoidance behavior despite analgesia	High anxiety/fear-avoidance predicts poor functional recovery; indicates need for graded exposure and behavioral activation	Asmundson and Katz (2009); Vlaeyen et al. (2016)
Emotional regulation capacity	Emotion Regulation Questionnaire (ERQ)/clinical interview	dlPFC, vlPFC, amygdala, insula	Effective reappraisal strengthens prefrontal-limbic control; maladaptive suppression increases central sensitization	Good emotional regulation predicts adaptive use of pain relief; deficits suggest benefit from mindfulness- or ACT-based interventions	Buhle et al. (2014), McCracken and Vowles (2014)
Coping style (active vs. passive)	Coping Strategies Questionnaire (CSQ); CISS	Executive Control Network, DMN	Active coping supports behavioral re-engagement and cortical reorganization; passive coping maintains disability loops	Passive coping predicts dissatisfaction despite pain reduction; active coping is a modifiable protective factor	Bernaerts et al. (2025), Brown and Nicassio (1987)
Locus of pain control	Beliefs about Pain Control Questionnaire (BPCQ)	dlPFC, ACC, reward network	Internal locus enhances engagement and learning-dependent neuromodulation effects	External locus predicts passivity and poor adherence; guides psychoeducation and motivational work	Crisson and Keefe (1988)
Illness acceptance	Acceptance of Illness Scale (AIS)	vmPFC, insula, DMN	Acceptance reduces affective amplification and rumination, facilitating functional gains	Higher acceptance predicts better post-implant adjustment; aligns with ACT framework	McCracken and Vowles (2014)
Cognitive status/executive functions	MoCA; FAB	dlPFC, fronto-parietal network	Executive deficits impair device management, learning, and adherence	Significant impairment may represent relative contraindication due to inability to manage SCS demands	Joyce et al. (2025), Lo Bianco et al. (2025)
Personality structure and response style	MMPI-2-RF	Distributed cortical–limbic systems	Somatization, defensiveness, and secondary gain reflect maladaptive brain–behavior patterns	Identifies psychological contraindications and need for tailored intervention	Doleys (2006), Goudman et al. (2022)

SCS, Spinal Cord Stimulation; CNS, Central Nervous System; PCS, Pain Catastrophizing Scale; PSEQ, Pain Self-Efficacy Questionnaire; BDI-II, Beck Depression Inventory, Second Edition; HADS, Hospital Anxiety and Depression Scale; TSK, Tampa Scale for Kinesiophobia; ERQ, Emotion Regulation Questionnaire; CSQ, Coping Strategies Questionnaire; CISS, Coping Inventory for Stressful Situations; BPCQ, Beliefs about Pain Control Questionnaire; AIS, Acceptance of Illness Scale; MoCA, Montreal Cognitive Assessment; FAB, Frontal Assessment Battery; MMPI-2-RF, Minnesota Multiphasic Personality Inventory-2, Restructured Form; dlPFC, dorsolateral prefrontal cortex; vlPFC, ventrolateral prefrontal cortex; vmPFC, ventromedial prefrontal cortex; ACC, anterior cingulate cortex; PAG, periaqueductal gray; RVM, rostral ventromedial medulla; NAc, nucleus accumbens; VTA, ventral tegmental area; DMN, default mode network.

### 7.2 Risk factors requiring evaluation

Several psychological and behavioral factors consistently predict poor SCS outcomes and warrant careful evaluation:

Cognitive impairment limits treatment comprehension, device operation, and adherence to rehabilitation protocols. Severe impairment typically excludes candidacy due to inability to provide informed consent and manage device programming (Lo Bianco et al., 2025).

Unrealistic expectations, particularly beliefs that SCS will completely eliminate pain, predict poorer outcomes (Goudman et al., 2022). Patients viewing SCS as a “cure” rather than pain management tool often report dissatisfaction even with clinically significant improvement.

Active substance use disorder compromises outcomes, with chronic high-dose opioid use associated with increased explantation risk (Wahezi et al., 2025). Stable recovery and appropriate medication management should precede implantation (Lo Bianco et al., 2025).

Treatment non-adherence, including missed appointments, medication non-compliance, or treatment abandonment, predicts similar difficulties with SCS’s intensive follow-up requirements for programming adjustments, battery management, and rehabilitation participation (Goudman et al., 2022; Shanthanna et al., 2023).

### 7.3 Protective factors to identify and Foster

Several psychological characteristics consistently predict favorable SCS outcomes. Clinicians should assess these protective factors and reinforce them as strengths supporting treatment success. Psychological resilience, including adaptive coping strategies and emotional regulation, supports successful adjustment to chronic pain and device management (Bernaerts et al., 2025). Active coping strategies, rather than passive approaches, predict greater recovery and satisfaction with SCS outcomes (Bernaerts et al., 2025).

## 8 Psychological optimization strategies

Evidence-based psychological interventions optimize modifiable risk factors and enhance SCS outcomes (Modak et al., 2023; Sparkes et al., 2012). SCS modulates supraspinal structures involved in cognitive-motivational aspects of pain processing, including limbic areas associated with pain affect and reward circuitry (De Ridder et al., 2013; Joosten and Franken, 2020). Maladaptive psychological patterns, particularly catastrophizing, are associated with altered cortical pain processing and poorer treatment outcomes across chronic pain interventions (Edwards et al., 2016a).

### 8.1 Pre-implantation CBT

Brief pre-operative CBT programs address modifiable risk factors through pain neuroscience education, cognitive restructuring, behavioral activation, and realistic expectation management (Ehde et al., 2014; Murphy et al., 2022; Sparkes et al., 2010). For high-risk patients, intensive psychological intervention may warrant deferring implantation until psychological optimization is achieved (Atkinson et al., 2011; Grider et al., 2016).

### 8.2 Acceptance and commitment therapy

Acceptance and Commitment Therapy (ACT) emphasis on accepting discomfort while pursuing valued activities aligns with realistic SCS expectations of meaningful but incomplete pain relief. Meta-analyses demonstrate medium-to-large effect sizes for ACT reducing depression, anxiety, pain catastrophizing, and psychological inflexibility while improving pain acceptance and functioning in chronic pain populations (Martinez-Calderon et al., 2024; Ye et al., 2024).

### 8.3 Mindfulness and emotion regulation

Mindfulness meditation demonstrates analgesic effects through altered attention, reduced catastrophizing, and enhanced emotional regulation. Brief mindfulness interventions of four 20-min sessions show significant pain-reducing effects, making mindfulness feasible within pre-implantation timelines (Zeidan et al., 2015).

### 8.4 Post-implantation follow-up

Structured follow-up during initial 3-6 months addresses adjustment challenges through motivational interviewing, problem-solving therapy, behavioral activation, cognitive restructuring, and relapse prevention. Technology-enhanced follow-up using mobile apps and telehealth platforms improves accessibility, patient engagement, and treatment outcomes (Gómez-González et al., 2025; Lee et al., 2022).

### 8.5 Interdisciplinary care models

Interdisciplinary pain rehabilitation programs integrating medical, psychological, physical therapy, and nursing expertise demonstrate superior outcomes compared to single-discipline approaches (Bean et al., 2025). Application to SCS involves coordinated team care throughout the treatment pathway.

## 9 Clinical translation: from neural mechanisms to actionable patient selection

While identifying neural correlates of psychological factors advances theoretical understanding, the critical question remains: how can this knowledge improve patient outcomes? This section translates the neurobiological framework into concrete clinical applications, demonstrating how understanding brain mechanisms transforms psychological screening from binary exclusion into dynamic, modifiable treatment targeting.

### 9.1 Contraindications and patient selection: distinguishing absolute, relative, and modifiable factors

Clinical decision-making requires distinguishing three categories: absolute contraindications precluding SCS, relative contraindications requiring management before proceeding, and modifiable risk factors addressable through intervention (Table 3).

**Table 3:** Clinical decision framework: contraindications, risk factors, and intervention pathways for spinal cord stimulation candidacy.

Category	Factor	Recommended action	Reassessment
Absolute medical/anatomical contraindications	Active infection	Exclude until resolved	After clearance (6–12 weeks)
	Uncontrolled coagulopathy	Exclude if bleeding risk unacceptable	Case-dependent
	Inadequate tissue coverage	Exclude	Not applicable
	Severe spinal canal stenosis	Exclude or decompress first	Post-decompression
Absolute psychological contraindications	Active psychosis	Defer; psychiatric treatment	After stabilization
	Active suicidal ideation	Defer; crisis intervention	≥3–6 months stability
	Active substance use disorder	Defer; addiction treatment	≥6–12 months remission
	Somatization / factitious disorder	Exclude	Not reconsidered
Relative contraindications	Severe depression (BDI > 30)	Defer; intensive treatment	BDI < 20
	Extreme catastrophizing (PCS > 40)	Defer; CBT	PCS < 30
	Cognitive impairment (no caregiver)	Defer or exclude	If support available
	Ongoing litigation	Consider deferral	After resolution
Modifiable psychological risk factors	Moderate catastrophizing (PCS 25–35)	CBT (6–8 weeks)	≥30% PCS reduction
	Moderate depression (BDI 15–25)	Behavioral activation ± meds	BDI < 14
	Pain anxiety (PASS 80–120)	CBT / ACT	PASS <80
	Low pain acceptance (CPAQ 30–45)	ACT	CPAQ >45
	Low self-efficacy (PSEQ 20–30)	Education, mastery training	PSEQ >30
	Unrealistic expectations	Psychoeducation	Expectations aligned

BDI, Beck Depression Inventory; PCS, Pain Catastrophizing Scale; PASS, Pain Anxiety Symptoms Scale; CPAQ, Chronic Pain Acceptance Questionnaire; PSEQ, Pain Self-Efficacy Questionnaire; CBT, Cognitive Behavioral Therapy; ACT, Acceptance and Commitment Therapy.

Absolute medical and anatomical contraindications include untreated infection, uncorrectable coagulopathy, inadequate tissue coverage, and anatomical barriers. These represent situations where the procedure poses unacceptable risk or is technically infeasible, as opposed to psychological factors (Dydyk and Tadi, 2023).

Absolute psychological contraindications include active untreated psychosis (precludes informed consent/programming), active suicidal ideation with plan/intent (requires urgent psychiatric intervention), current substance use disorder (alters pain perception and device management), and somatization/factitious disorder (pain serves psychological needs not amenable to neuromodulation) (Atkinson et al., 2011; Fisher et al., 2024).

Relative contraindications require deferral pending appropriate intervention. For example, severe refractory depression (BDI-II > 28 despite treatment) necessitates cognitive behavioral therapy and psychiatric care before SCS can be considered (Modak et al., 2023). Extreme catastrophizing shows conflicting evidence: some studies report PCS ≥ 30 correlates with poorer outcomes and five-fold increased dissatisfaction (Rosenberg et al., 2015), while others found no association between baseline PCS and long-term results (Poulsen et al., 2021; Sanapati et al., 2024). Given this controversy, extreme catastrophizing (PCS > 40) resistant to adequate CBT warrants intensive intervention but should not constitute absolute exclusion (Sparkes et al., 2012). Significant cognitive impairment without capable caregivers precludes treatment unless adequate support systems exist (Atkinson et al., 2011); severe impairment preventing informed consent represents exclusion (Fisher et al., 2024; Lo Bianco et al., 2025). Active pain-related litigation warrants deferral due to potential secondary gain and outcome confounding (Kumar et al., 2006; Mehta et al., 2025). These conditions warrant deferral for intensive intervention (3-6 months), with reassessment determining ultimate candidacy (Atkinson et al., 2011; Mehta et al., 2025).

Modifiable risk factors warrant pre-implant optimization through targeted interventions. Moderate catastrophizing (PCS 25-35), mild-to-moderate depression (BDI 15-25), anxiety, low pain acceptance (CPAQ low scores), and modest self-efficacy concerns (PSEQ ≤18-20) should be addressed with 6-12 week targeted interventions prior to implantation (Bendinger et al., 2015; Mehta et al., 2025). These psychological factors predict suboptimal outcomes but remain amenable to modification through CBT, pain education, and acceptance-based interventions (Molloy et al., 2006; Sparkes et al., 2012). Low pain self-efficacy (PSEQ ≤18) represents a specific risk factor for treatment failure (Bendinger et al., 2015), while individuals with low acceptance demonstrate greater catastrophizing and depressive symptoms (Azuero et al., 2024). Improvement of ≥30% on relevant measures following targeted intervention indicates sufficient optimization; lack of response may necessitate reclassification as relative contraindication requiring more intensive multidisciplinary management (Atkinson et al., 2011; Mehta et al., 2025).

The decision algorithm follows a stepwise approach: First, screen for absolute medical and anatomical contraindications; exclude candidates if present. Second, screen for absolute psychological contraindications; defer pending psychiatric stabilization. Third, assess relative contraindications; defer 3-6 months for intensive treatment. Fourth, identify modifiable risk factors; implement 6-12 week interventions and reassess. Finally, proceed with implantation when risk factors are adequately addressed.

The goal is a balanced approach that avoids both inappropriate exclusion and inappropriate inclusion through individualized assessment, targeted intervention, and shared decision-making informed by realistic prognosis based on each patient’s psychological profile.

### 9.2 From brain circuits to bedside decisions

Integration of neuroimaging data with psychological assessment creates a mechanistic roadmap for intervention. Rather than simply identifying that catastrophizing predicts poor outcomes, we now understand this operates through anterior cingulate cortex and anterior insula hyperactivation during pain processing (Seminowicz and Moayedi, 2017). This enables targeted interventions: therapies normalizing anterior cingulate cortex function (cognitive-behavioral approaches, mindfulness training) become rationally rather than empirically selected.

Consider a patient scoring highly on the PCS (PCS > 30), traditionally a negative prognostic indicator (Rosenberg et al., 2015). Without mechanistic understanding, clinicians face binary choice: proceed despite poor prognosis or exclude from treatment. The neurobiological framework offers a third option: targeted pre-implantation intervention. Knowing catastrophizing reflects modifiable anterior cingulate cortex hyperreactivity (Gracely et al., 2004; Seminowicz and Davis, 2006), clinicians recommend brief CBT (6-8 sessions) targeting catastrophic cognitions (Seminowicz et al., 2013). Post-intervention reassessment determines whether sufficient neural and psychological readiness has been achieved (Gibson and Sabo, 2018; Mehta et al., 2025).

### 9.3 Multi-level assessment and intervention framework

We propose a stratified approach integrating psychological assessment with neural mechanistic understanding:

Level 1: Standard Psychological Screening - All patients undergo validated assessment:

- Pain Catastrophizing Scale (PCS)
- Chronic Pain Acceptance Questionnaire (CPAQ)
- Pain Self-Efficacy Questionnaire (PSEQ)
- Beck Depression Inventory (BDI)
- Pain Anxiety Symptoms Scale (PASS).

Level 2: Mechanistic Risk Stratification - Results interpreted through neurobiological framework:

- High catastrophizers (PCS > 30): anterior cingulate cortex /insula hyperactivation requires cognitive reappraisal interventions,
- Low pain acceptance (CPAQ < 40): Excessive prefrontal cortex-amygdala coupling benefits from acceptance-based interventions,
- Compromised reward function (BDI > 20, anhedonia): Mesolimbic dysfunction requires interventions enhancing reward sensitivity,
- Executive dysfunction (TMT B-A > 60s): dorsolateral prefrontal cortex compromise necessitates cognitive rehabilitation or simplified protocols.

Level 3: Targeted Pre-Implantation Interventions:

- CBT for Pain (6-8 sessions): Targets catastrophic cognitions and anterior cingulate cortex hyperreactivity,
- Acceptance and Commitment Therapy (8-10 sessions): Enhances psychological flexibility and prefrontal regulation,
- Behavioral Activation (8-12 sessions): Primes mesolimbic reward circuitry,
- Mindfulness interventions (8 weeks): Modulates anterior cingulate cortex reactivity and enhances prefrontal control,
- Expectation management: Structured education leveraging dorsolateral prefrontal cortex circuits.

### 9.4 Proposed pre-implantation neurophysiological protocol (PINP): integrating electrodiagnostic and neurophysiological biomarkers

Psychological assessment alone is insufficient to determine neuromodulation readiness, as effective SCS also requires an intact peripheral nociceptive substrate, functional descending inhibition, and cortical capacity for neuroplastic reorganization. The PINP therefore adds two neurophysiological assessment tiers to the existing psychological framework: a neurological prerequisite applied to all candidates (Level 0) and an extended evaluation reserved for high-risk patients identified at Level 2 (Table 4).

**Table 4:** Pre-implantation neurophysiological protocol (PINP): assessment methods, indices, and clinical decision thresholds for SCS candidacy.

Method	What it assesses	Key index	Risk threshold	Clinical implication
ENG/NCS	Peripheral nerve integrity; confirmation of neuropathy type	SNAP/CMAP amplitude; conduction velocity; denervation signs on needle EMG	Absent SNAP, or amplitude <50% LLN; active denervation on EMG	Severe axonal loss; may independently preclude SCS response regardless of psychological profile; multidisciplinary review required
SSEP	Integrity of ascending large-fibre somatosensory pathways (dorsal column–medial lemniscal system to cortex); substrate for supraspinal SCS modulation	Cortical N20 (upper limb)/P40 (lower limb) amplitude and latency	Absent or severely attenuated cortical response	Large-fibre pathway dysfunction; SCS efficacy limited regardless of stimulation parameters; prolonged central conduction time associated with poorer long-term SCS outcomes; defer/reconsider
LEP (where available)	Integrity of Aδ and C fibre nociceptive pathways and spinothalamic tract conduction from periphery to cortex; complementary to SSEP when small-fibre or spinothalamic pathology is suspected	N2–P2 amplitude and latency (vertex complex)	Absent or severely attenuated N2–P2; significantly prolonged latency relative to normative values	Spinothalamic tract dysfunction; consider when SSEP is normal but nociceptive pathway integrity is in doubt; particularly relevant in PDPN, CRPS, and postherpetic neuralgia
CHEP (alternative to LEP)	Aδ fibre function and spinothalamic tract integrity via contact heat stimulation; clinically practical non-invasive alternative when laser is unavailable	N2–P2 amplitude and N2 latency	Absent or markedly reduced N2–P2 amplitude relative to age- and sex-adjusted normative values	Small-fibre neuropathy or spinothalamic dysfunction; informs candidacy in conditions not captured by standard SSEP or ENG/NCS
Static QST *(DFNS protocol)*	Somatosensory function across all fibre types; detection and pain thresholds; sensory loss and gain phenomena (allodynia, hyperalgesia, paradoxical heat sensations)	Thermal and mechanical detection and pain thresholds; z-scores relative to DFNS normative values	Marked sensory loss or gain pattern inconsistent with stated pain distribution; mechanistic subtype identification	Profiles neuropathic pain subtype; supports stratification according to differential analgesic response potential
Dynamic QST – CPM	Endogenous descending inhibitory capacity (PAG–RVM axis); functional analogue of supraspinal SCS target	CPM effect (ΔNRS or ΔPPT during cold water immersion conditioning, 8–12 °C)	CPM effect <10% (absent or negligible inhibition); lab-dependent threshold	Deficient descending inhibition; among the strongest predictors of attenuated neuromodulatory response; consider adjunct or combined neuromodulation strategies
Dynamic QST – TS	Spinal cord excitability via wind-up; central sensitization at dorsal horn level	TS slope (ΔNRS across repeated 1 Hz stimuli); wind-up ratio	Significant positive slope across stimulus series; wind-up ratio >2 (DFNS protocol reference)	Central sensitization present; supraspinal modulation alone may be insufficient; address central mechanisms prior to or alongside SCS

ENG, electroneurography; NCS, nerve conduction studies; SNAP, sensory nerve action potential; CMAP, compound muscle action potential; LLN, lower limit of normal; SSEP, somatosensory evoked potentials; LEP, laser evoked potentials; CHEP, contact heat evoked potentials; QST, quantitative sensory testing; CPM, conditioned pain modulation; TS, temporal summation; NRS, numerical rating scale; PPT, pressure pain threshold; PAG, periaqueductal gray; RVM, rostral ventromedial medulla; PDPN, painful diabetic peripheral neuropathy; CRPS, complex regional pain syndrome; DFNS, German Research Network on Neuropathic Pain; PCS, Pain Catastrophizing Scale; BDI, Beck Depression Inventory; SCS, spinal cord stimulation.

Level 0 (Neurological prerequisite – all candidates): Electroneurography and nerve conduction studies (ENG/NCS) confirm the presence, type, and severity of peripheral sensory nerve injury prior to psychological screening. Severe axonal loss may independently preclude a meaningful neuromodulation response regardless of psychological profile (De Vos et al., 2014; Slangen et al., 2014). Sensory ENG/NCS is established practice in centers evaluating SCS candidates for painful diabetic peripheral neuropathy, radiculopathy, and CRPS II, and its formal inclusion as a prerequisite step aligns the protocol with existing clinical guidelines (Atkinson et al., 2011; Shanthanna et al., 2023).

Extended neurophysiological assessment (high-risk patients identified at Level 2): Patients meeting one or more high-risk criteria at Level 2 (PCS > 30, BDI > 20, suspected central sensitization, or severe neuropathy on ENG/NCS) undergo additional objective assessments.

Somatosensory evoked potentials (SSEP) evaluate the integrity of ascending large-fiber somatosensory pathways from the peripheral nerve through the dorsal column–medial lemniscal system to the cortex. Absent or severely attenuated cortical SSEP components suggest large-fiber pathway dysfunction that may limit SCS efficacy irrespective of stimulation parameters (Loeser and Treede, 2008). The clinical relevance of preoperative SSEP assessment has been demonstrated empirically: in a 95-patient cohort followed over a mean of 18.8 months, prolonged central conduction time on preoperative SSEP was significantly associated with poorer long-term SCS outcomes (Sindou et al., 2003).

It should be noted, however, that SSEP does not capture small-fiber or spinothalamic tract integrity. Since the spinothalamic tract constitutes the primary ascending pathway for pain and temperature sensation, its functional status is of direct mechanistic relevance to SCS candidacy assessment. Laser evoked potentials (LEP) provide objective electrophysiological evaluation of Aδ and C fiber function and spinothalamic tract conduction from the periphery to the cortex, and are currently regarded as the most reliable neurophysiological tool for assessing nociceptive pathway integrity (Treede et al., 2003). Where laser stimulation is unavailable, contact heat evoked potentials (CHEP) represent a clinically practical, non-invasive, and objective alternative for small-fiber assessment (Atherton et al., 2007; Granovsky et al., 2016). Separate evaluation using LEP or CHEP should therefore be considered in all SCS candidates presenting with pain conditions in which small-fiber or spinothalamic pathology is clinically suspected (Atherton et al., 2007). In addition, quantitative sensory testing (QST), following the standardized protocol of the German Research Network on Neuropathic Pain (DFNS), profiles somatosensory function across all fiber types, identifying sensory loss and gain phenomena that may reflect the mechanistic subtype of neuropathic pain and predict differential analgesic response (Baron et al., 2017). Conditioned pain modulation (CPM) assesses descending inhibitory capacity via cold water immersion (8–12 °C); a CPM effect below 10% indicates deficient PAG–RVM inhibition and is among the strongest predictors of attenuated neuromodulatory response (Nurmikko et al., 2024; Yarnitsky, 2015). Temporal summation (TS) quantifies spinal wind-up via repeated stimuli at 1 Hz; a steep TS slope indexes dorsal horn central sensitization and may signal insufficient supraspinal modulation (Lütolf et al., 2022). SCS has been shown to restore CPM efficiency and attenuate temporal summation in responders, supporting the use of baseline psychophysical profiles as both selection and monitoring biomarkers (Kriek et al., 2023).

PINP results are synthesized into a Neuromodulation Readiness profile with three outcomes. “Proceed” (no tier at risk threshold) supports standard implantation. “Conditional” (one or two tiers at threshold) triggers a 6–12 week prehabilitation period before reassessment. “Defer/Reconsider” (severe axonal loss on ENG/NCS with absent CPM and/or SSEP) requires multidisciplinary review and consideration of alternative pain management. Neurophysiological findings contextualize psychological risk within the biological reality of each patient’s nervous system, shifting selection from a binary decision into a precision-guided, modifiable process.

## 10 Future directions and research gaps

Despite robust evidence linking psychological factors to SCS outcomes through identifiable neural mechanisms, translating this understanding into clinical practice requires addressing critical gaps. Future advances should focus on objective measurement, precision prediction, and novel intervention strategies.

### 10.1 Neurophysiological biomarkers and combined neuromodulation

EEG and TMS-EEG offer lower cost, greater accessibility, and feasibility for repeated assessment. EEG signatures index psychological vulnerabilities: frontal alpha asymmetry reflects reward sensitivity (Cavanagh and Shackman, 2015); error-related negativity characterizes anxiety and catastrophizing (Cavanagh and Shackman, 2015); reward positivity predicts benefit capacity (Proudfit, 2015); theta power correlates with catastrophizing, while alpha/beta ratios index chronic pain connectivity patterns (Camfferman et al., 2017; Stern et al., 2006). TMS-EEG probes cortical plasticity: preserved plasticity predicts better neuromodulation response, while rigidified networks predict poorer outcomes (De Martino et al., 2025). Since psychological factors operate through cortical circuits (anterior cingulate cortex, dorsolateral prefrontal cortex, insula, nucleus accumbens) unaffected by spinal stimulation, combining SCS with transcranial direct current stimulation (tDCS) offers mechanistic rationale. Dorsolateral prefrontal cortex stimulation enhances executive function and reduces catastrophizing (Alcon et al., 2025; Carvalho et al., 2018; Marcinkowska et al., 2025), while M1 stimulation modulates sensorimotor processing (O’Connell et al., 2018). Home-based tDCS produces effect sizes (d = 0.4-0.6) comparable to pharmacological interventions with superior tolerability (Carvalho et al., 2018; Fregni et al., 2021). The framework extends beyond chronic pain: in spinal cord injury (SCI), where 50-80% experience neuropathic pain with psychological comorbidity operating through identical neural circuits (Burke et al., 2017; Peterson et al., 2022), combined SCS-tDCS may enhance cognitive-emotional regulation alongside spinal modulation (Herrity et al., 2018; Kapadia et al., 2020).

### 10.2 Multimodal predictive models

Machine learning approaches identify complex interactions among psychological measures, neurophysiological markers, and clinical variables that traditional methods cannot capture (Gopal et al., 2025; Prunskis et al., 2025). Integration of electroencephalographic biomarkers with multimodal clinical data enables precision matching of patients to optimal neuromodulation approaches (Gopal et al., 2025; Hadanny et al., 2022).

### 10.3 Digital health technologies

Virtual reality (VR) combined with SCS demonstrates enhanced efficacy, with personalized multisensory feedback producing 44% pain reduction versus 23% with non-personalized approaches (Solcà et al., 2021). VR enables immersive exposure therapy for fear-avoidance, mindfulness practice, and real-time biofeedback. Smartphone-based ecological momentary assessment captures real-time dynamics invisible to retrospective reporting. Telehealth platforms expand access to psychological services with efficacy comparable to in-person care (Herbert et al., 2017).

## 11 Conclusion

Spinal cord stimulation (SCS) is an evidence-based therapy for refractory neuropathic pain, yet outcomes remain highly variable despite technological advances. The evidence reviewed here indicates that SCS efficacy is not determined by device parameters alone: durable benefit depends on the functional state of supraspinal networks that govern pain appraisal, motivation, learning, and the ability to translate analgesia into functional recovery.

Across studies, key psychological predictors—pain catastrophizing, depression, anxiety/fear-avoidance, low self-efficacy, passive coping, unrealistic expectations, and limited social support—align with identifiable neural mechanisms. Maladaptive profiles are linked to reduced prefrontal top-down control and heightened salience/limbic reactivity (anterior cingulate cortex, insula, amygdala), impaired reward valuation and reinforcement learning (mesolimbic circuitry), and persistent central sensitization. Conversely, adaptive factors (realistic expectations, perceived control, emotion regulation, active coping, supportive context) facilitate coordinated engagement of prefrontal–limbic–brainstem circuits supporting descending inhibition and neuroplastic reorganization. Incorporating cerebellar contributions strengthens this model by adding predictive coding and associative learning mechanisms that plausibly connect catastrophizing and fear-avoidance with persistent pain and reduced responsiveness to neuromodulation.

Clinically, psychological screening should move beyond binary risk stratification toward identifying modifiable targets that determine neuromodulation readiness. A practical pathway is a stepped approach: standardized multidomain assessment, differentiation of absolute vs. relative vs. modifiable factors, and targeted pre-implant psychological prehabilitation (expectancy shaping, CBT/ACT, behavioral activation, graded exposure for fear-avoidance, and emotion regulation training). This is mechanistically justified because these interventions engage the same networks that SCS relies on—agency-related prefrontal control, reward-based reinforcement of relief, and learning-dependent re-engagement in activity.

Future progress requires multimodal predictive models integrating psychological phenotypes with objective biomarkers (e.g., EEG/TMS-EEG measures of oscillatory balance and cortical excitability), alongside digitally supported and combined neuromodulation strategies that address spinal and cortical mechanisms in parallel. Overall, psychological readiness should be viewed as a neurobiological prerequisite for SCS efficacy, and its systematic optimization offers a direct route to reducing outcome variability and improving long-term functional recovery.
